# 

**Published:** 1887-03-05

**Authors:** 


### THE HOSPITAL

An Institution, Family, and Parochial Journal of Hospitals, Asylums, & all Agencies for the Care of the Sick, Criticism & News.

### CONTENTS.

**Nurses' Recreations** - - - - - - 379

**Words of Consolation.**—XXIII. Nurses' Trials—Temper - - - - - - - 380

**Nursing Institutions and Hospitals.** The Case for the Private Institutions and Nurses. By Mrs. E. A. Bedingfeld, Miss Meyrick, and a Trained Nurse - 381

**In the Children's Ward.** By Walter J. Eccott. Chap. I - - - - - - - 383

**Farthing Dinners.** By a Young Hand - - 384

**Notes and News.**—Miscellaneous Items.—Madame Roze at the Jaffray Hospital.—Entertainment at Lowther Lodge.—The Seamen's Hospital.—Vacancies.—The Stroud General Hospital.—The British Hospital at Port Said.—Donations and Legacies to Medical Charities.—The Macclesfield Infirmary.—The Lord Provost of Glasgow and the Hospitals, etc., etc. - - - - 386

**Annotations.**—Miss Manson and Trained Nurses.—Drinks Wanted.—Who stands Sponsor for the Matrons? 389

**Chats about Medical Museums.** By One of the Crowd - - - - - - - 390

**Till the Doctor Comes.** XII. Minor Injuries, etc. 391

**Our Skin, and How to Take Care of it** - - 391

**Hospital Huts** - - - - - - 392

**Incidents of Animal Life.** Collated by the Rev. F. O. Morris, B.A. - - - - - 392

**Doctors Described by Nurses and Patients** - 393

**The Editor's Letter-Box.**—

Infectious *v.* Non-Infectious Convalescents - - 393

"Incubators for Infants" - - - - - 393

**In- and Out-Patients' Hospital Diary** - - 394

**The Children's Corner.**—Puzzles Answers, etc. - v

